# Laminin-Mediated Interactions in Thymocyte Migration and Development

**DOI:** 10.3389/fimmu.2015.00579

**Published:** 2015-11-17

**Authors:** Wilson Savino, Daniella Arêas Mendes-da-Cruz, Daiane Cristina Ferreira Golbert, Ingo Riederer, Vinicius Cotta-de-Almeida

**Affiliations:** ^1^Laboratory on Thymus Research, Oswaldo Cruz Institute, Oswaldo Cruz Foundation, Rio de Janeiro, Brazil

**Keywords:** thymus, T-cell development, T-cell migration, thymic epithelial cells, extracellular matrix, laminin isoforms, integrins, chemokines

## Abstract

Intrathymic T-cell differentiation is a key process for the development and maintenance of cell-mediated immunity, and occurs concomitantly to highly regulated migratory events. We have proposed a multivectorial model for describing intrathymic thymocyte migration. One of the individual vectors comprises interactions mediated by laminins (LMs), a heterotrimeric protein family of the extracellular matrix. Several LMs are expressed in the thymus, being produced by microenvironmental cells, particularly thymic epithelial cells (TECs). Also, thymocytes and epithelial cells express integrin-type LM receptors. Functionally, it has been reported that the *dy/dy* mutant mouse (lacking the LM isoform 211) exhibits defective thymocyte differentiation. Several data show haptotactic effects of LMs upon thymocytes, as well as their adhesion on TECs; both effects being prevented by anti-LM or anti-LM receptor antibodies. Interestingly, LM synergizes with chemokines to enhance thymocyte migration, whereas classe-3 semaphorins and B ephrins, which exhibit chemorepulsive effects in the thymus, downregulate LM-mediated migratory responses of thymocytes. More recently, we showed that knocking down the ITGA6 gene (which encodes the α6 integrin chain of LM receptors) in human TECs modulates a large number of cell migration-related genes and results in changes of adhesion pattern of thymocytes onto the thymic epithelium. Overall, LM-mediated interactions can be placed at the cross-road of the multivectorial process of thymocyte migration, with a direct influence *per se*, as well as by modulating other molecular interactions associated with the intrathymic-trafficking events.

## Introduction

**Figure 1:**
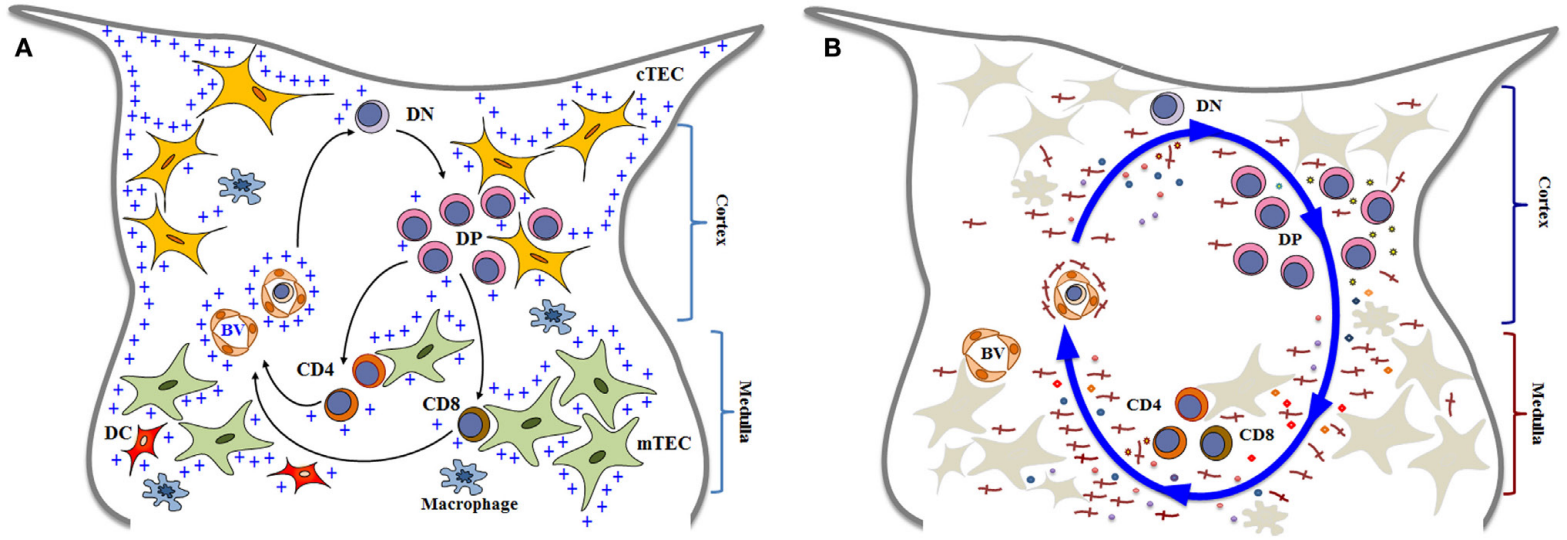
**Simplified diagrams of thymocyte differentiation and migration, in the context of the thymic microenvironment, according to a multivectorial migratory pattern**. In **(A)**, we show that bone marrow-derived T-cell precursors enter the thymus through blood vessels (BV) in the corticomedullary junction of thymic lobules. CD4^−^CD8^−^ double negative (DN) immature thymocytes then migrate to the outer cortex, where they interact with cortical thymic epithelial cells (cTEC) and differentiate into CD4^+^CD8^+^ double positive (DP) cells. These cortical thymocytes will be able to continue to interact with microenvironmental cells as they migrate to the medulla, and positively selected cells will become either CD4 single-positive (CD4 SP) or CD8 single-positive (CD8 SP) cells, by interacting with medullary TEC (mTEC) or dendritic cells (DC) the medulla. In addition to cell–cell interactions between thymocytes and the microenvironmental components, developing thymocytes can be influenced by components of the extracellular matrix (herein labeled with blue crosses). Mature SP thymocytes will leave the organ by entering blood vessels that will allow them to find the way to colonize the T-cell-dependent zones of peripheral lymphoid organs. Although not showed in the cartoon, most thymocytes actually die and are rapidly phagocytized by macrophages. In **(B)**, we illustrate that thymocyte migration occurs as a result of multiple interactions, following a multivectorial pattern throughout the thymic lobule. In this panel, we emphasize the importance of LM (the red crosses) as one vector involved in thymocyte migration, comprising both immature and mature cells. Diagrams modified from Ref. (16, 18, 81, 128, 129).

The thymus plays a central role on the immune system, as the primary lymphoid organ where T ­lymphopoiesis takes place (1, 2). It is responsible for generating lymphocytes that exhibit a diverse group cells expressing the T-cell receptor (TCR), including the conventional CD4^+^ and CD8^+^ TCRαβ T cells, TCRγδ T cells, Foxp3^+^ regulatory T cells, and the invariant NK T cells (3). Along with this whole process, the thymus stems a complex set of differentiating signals that target bone marrow-derived lymphoid progenitors which settle the thymus to become committed to T-cell lineage and further originating those distinct T-cell lineages (4). In this context, intrathymic T-cell ­differentiation is considered a key process for the development and maintenance of cell-mediated immune responses. Accordingly, bone marrow-derived T-cell precursors enter the thymus where they undergo a complex series of biological events, so that around 95% of the cells generated within the organ die by apoptosis. Meanwhile, a small proportion of cells is rescued from programed cell death and acquires the ability to exit the organ, ultimately reaching the thymus-dependent areas of the secondary lymphoid organs Figure 1A [reviewed in Ref. (5–7)]. These mature T cells are then able to mount a cell-mediated immune response.

The developmental journey that generates the conventional CD4^+^ and CD8^+^ αβ T cells has been extensively studied and involves a series of ordered proliferative and selective events, under the central influence of gene recombination at the TCR loci, that results in pre-TCR and αβ TCR assembling (8). The pathway starts after progenitor cells enter the thymus at the so-called corticomedullary junction and migrate from the cortex in the direction to the subcapsular area as cells lacking CD4 and CD8 coreceptors, defined as double-negative (DN; CD4^−^CD8^−^) thymocytes. Here, the TCR rearrangement is tested through the expression of a pre-TCR, in a process called β-selection (9). Following the TCRα chain assembly at the highly expanded double-positive (DP; CD4^+^CD8^+^) stage, TCR specificity is tested in a process known as positive selection, where low-avidity interactions with self peptide–MHC complexes expressed on thymic epithelial cells (TECs) allow survival and further differentiation (10, 11). The differentiating DP thymocytes start to lose the expression of either CD4 or CD8 and migrate from the cortex toward the thymic medulla. The pathways leading to CD4 or CD8 SP subsets are dependent on distinct transcriptional machineries, seem to initiate at the transitional stage characterized as CD4^+^CD8^lo^, and depend on the TCR interaction with MHC class II or class I [reviewed in Ref. (12–14)].

The most critical developmental cues arise from the tissue microenvironment. In this context, thymic microenvironmental cells – comprising cortical and medullary TECs as well as other stromal cells of mesodermal origin – provide the milieu for T-cell development and the final selection of a proper set of the T-cell repertoire (15).

The microenvironmental signals include cell surface and soluble molecules, such as Delta-class Notch ligand Delta-like 4 (*Dll4*), kit ligand, MHC molecules, and interleukin-7 (IL-7). Likewise, as intrathymic T-cell differentiation occurs concomitantly to highly regulated migratory events, chemokine-derived signals are present from the progenitor entrance until the exit of mature SP cells for the periphery (7, 16, 17). In fact, as discussed below, we have proposed a multivectorial model for describing intrathymic migration of developing thymocytes (18). In this context, interactions with the extracellular matrix (ECM) also critically operate throughout the intrathymic T-cell maturational pathway (19).

Thymic epithelial cells are the major cellular component of the thymic microenvironment and are located in both the cortex (cTEC) and medulla (mTEC) of the thymic lobules. A special lymphoepithelial complex named thymic nurse cells (TNC) is located in the outer cortex and can be used as an *in vitro* model to study thymocyte migration in a tridimensional TEC context (16). In the medulla, a particular mTEC component is able to express the transcription factor autoimmune regulator which controls the thymic expression of so-called tissue-specific antigens, and this interaction is important for avoiding autoimmunity (20, 21).

As seen below, microenvironmental cells are responsible to produce the ECM network in the thymus, as well as other cell migration-related moieties, such as chemokines (16, 19).

In this review, we specifically highlight the role of laminin (LM) isoforms for intrathymic T-cell migration and maturation in both physiological and pathological conditions.

## Intrathymic Expression of LM Isoforms and Integrin-Type LM Receptors

Laminins correspond to a family of heterotrimeric ECM proteins formed by an α, β and γ chain; each being encoded by a specific gene. Several LM isoforms have been described and are classified according to the corresponding α, β, and γ chains. Once synthesized, the chains form a cross-shaped coiled-coil structure. Currently, five α, three β, and three γ chains have been chemically characterized, and 18 isoforms reported (see Table 1). The current nomenclature uses Arabic numerals based on the three chain numbers. For example LM-111 is formed by α1, β1, and γ1 chains, whereas LM-211 is composed by α2, β1, and γ1 chains (22–24).

**Table 1:** **Laminin isoforms and corresponding integrin-type laminin receptors^a^**.

Laminin isoforms		Composition (α, **β**, and **γ** chains)	Preferential integrin receptors
Present nomenclature	Previous nomenclature		
LM-111	Laminin-1	α1β1γ1	α1β1, α2β1, α3β1 α6β1, α6β4, and α7β1
LM-121	Laminin-3	α1β2γ1	α6β1, α6β4, and α7β1
LM-211	Laminin-2/merosin	α2β1γ1	α6β1, α3β1, α6β4, and α7β1
LM-212	–	α2β1γ2	α3β1, α6β1, and α7β1
LM-222	–	α2β2γ2	α3β1, α6β1, and α7β1
LM-213	Laminin-12	α2β1γ3	–
LM-221	Laminin-4	α2β2γ1	α7β1
LM-311	Laminin-6/Laminin-6A	α3β1γ1	α3β1 and α6β1
LM-321	Laminin-7/laminin-7A	α3β2γ1	ND
LM-323	Laminin-13	α3β2γ3	–
LM-332	Laminin-5	α3Bβ2γ1	α1β1, α3β1, α6β1, and α6β4
LM-411	Laminin-8	α4β1γ1	α3β1, α6β1, and α7β1
LM-421	Laminin-9	α4β2γ1	α6β1
LM-423	Laminin-14	α4β2γ3	–
LM-511	Laminin-10	α5β1γ1	α3β1, α6β1, and α6β4
LM-521	Laminin-11	α5β2γ1	α3β1, α6β1, and α6β4
LM-522	–	α5β2γ2	α3β1
LM-523	Laminin-15	α5β2γ3	–

*^a^Data adapted from Ref. (25–40)*.

*ND, not determined; – absence of integrin binding because of the γ3 chain of the LM isoform (27)*.

Laminin-mediated interactions are triggered by two main classes of receptors, integrin- and non-integrin-type receptors (41, 42). At least eleven integrins, namely α1β1, α2β1, α2β2, α3β1, α6β1, α6β4, α7β1, α9β1, αvβ3, αvβ5, αvβ8, and αMβ2, can bind to LMs (25, 43–49). Moreover, there is, to some extent, a specificity of given integrins to bind different LMs. For example, the integrins α6β1 and α3β1 (CD49f/CD29 and CD49c/CD29, respectively) recognize both LM-511 and LM-521 (26). A second integrin bearing the α6 subunit, the α6β4 LM receptor (CD49f/CD104), presents the highest affinity for LM-3A32 (26).

Upon LM, binding integrins acts through outside-in signaling, promoting modulation in phosphorylation states and activities of cytosolic tyrosine kinases, such as focal adhesion kinase and Src family kinases, which subsequently regulate other kinases, scaffolding proteins, and intracellular signal transduction (50, 51). Such signaling is initially triggered by the binding of the globular domains of LM α-chains to integrins (24). In this respect, LM isoforms containing the γ3 chain are unable to bind to integrins due to the absence of the glutamic acid residue, which is conserved in the C-terminal regions of the γ1 and γ2 chains (27).

The list of LM isoforms and corresponding integrin-type receptors so far described is summarized in Table 1, and the general gene and protein features, as well as functions in the hemopoietic system can be fully accessed at the LM Database Version 2.0: www.lm.lncc.br (28).

Laminins are heterogeneously distributed within the thymic lobules. The initial immunolocalization studies, using anti-LM polyclonal antibodies that do not discriminate a specific LM isoform, revealed a heterogeneous distribution, with the medulla containing a denser LM network as compared to the cortex (52). Additionally, perivascular spaces (PVS) as well as periseptal basement membranes contain LM. This distribution pattern is observed in both human and mouse thymuses (52–54). Moreover, *in vitro* studies revealed that TECs, as well as phagocytic cells of the thymic reticulum, are able to produce LM (52, 53, 55).

Gene expression analyses revealed that genes coding for several LM chains are constitutively expressed in the human thymus (29, 56, 57). Moreover, taking advantage of the availability of antibodies able to specifically discriminate LM chains, isoforms could be more precisely located within the thymus.

Laminin-111 appears early and is the main LM isoform during embryogenesis (58–60). However, in adults, LM-111 expression is very limited, being detected in some epithelial basement membranes (61, 62). Interestingly, although PCR amplification from bulk thymus extracts did not reveal the α1 LM chain (29, 57), it was clearly identified in extracts obtained from cultured TEC or TNC (57). This result points to the possibility that particular culture conditions may modify the expression of LM chains. Moreover, In addition to its role in thymocyte migration (further discussed below), LM-111 is able to stimulate the proliferation both mouse and human TEC (53, 63).

Laminin-211 (formerly called merosin) is present in the basement membrane of the skeletal muscle (64) and also occurs in different organs, including the mouse and human thymus (29, 30, 57). The α2 LM chain is observed in the subcapsular epithelium and blood vessels of human thymus (29); being also detected in cultures of TEC and TNC complexes (57). In mice, immature thymocytes bind more to LM-211 when compared to mature ones, and use the α6β1 integrin, rather than α6β4 to adhere to this isoform (30). Human thymocytes express both α6β1 and α3β1 integrin-type LM receptors, which mediate the binding to this isoform and are involved in thymocyte proliferation triggered by a costimulatory signal from anti-CD3 antibody plus LM-211 (31). However, differently from these results, it has been reported that none of the thymocyte subpopulations bind to LM-211 or LM-221 (29). Such discrepancy may be due to different experimental conditions, or even the quality of the LM preparations.

However, *in vivo* data revealed that mice spontaneously lacking LM-211 (*dy/dy*) show a disorganized and smaller thymus, with a reduced number of thymocytes, particularly in DP subpopulation. In these animals, capsular and subcapsular DN thymocytes exhibit increase in apoptosis (65). Using another α2 LM mutant mouse (*dy3k/dy3k*), an apoptosis-related decrease in the DP subset was also reported. Furthermore, LM-211 increased the viability of normal thymocytes (66). Interestingly, the lack of LM-211 in the animals also compromises the functional innervation of the organ, since the acetylcholinesterase activity of the *dy/dy* mouse thymus is decreased in 50% as compared to age/matched normal (67). Taken together, these data indicate a pleiotropic effect of LM-211 upon the thymus.

Studies on the distribution of LM-332, using reagents able to define the localization of α3, β3, and γ2 LM chains, revealed that such isoform is present in the medullary area of the thymic parenchyma and the basal laminae of the subcapsular cortex and of blood vessels. More precisely, LM-332 was localized in epithelial cells (defined by keratin staining) and surrounding blood vessels, including capillaries (29, 56). Three-dimensional reconstruction of human thymic lobules, using confocal microscopy after immunohistochemical staining revealed a sort of LM-332-containing conduit structure surrounded by TECs in the medulla of the thymic lobules. In these conduits, immunohistochemistry staining with polyclonal antibodies for LM from Engelbreth-Holm-Swarm murine sarcoma basement membrane, which do not recognize LM-332, indicates that other LM isoforms can be present. Indeed, TEC primary cultures, but not thymic dendritic cells that also surround the conduits, express the LM chains containing the isoform 332. Measurements of these structures revealed that they are too narrow to allow a flow of cells, thus raising the hypothesis that they provide an influx of molecules in the thymic medulla (68). Yet, experimental evidence to prove this possibility remains to be obtained.

Functionally, it has been shown that soluble LM-332 inhibits human thymocyte proliferation induced by anti-CD3 plus IL-2 (56). In another work, CD8^+^ SP thymocytes were the only subpopulation able to adhere onto LM-332, being this adhesion dependent of α6β4 integrin (29). These results indicate that this isoform can be involved in the differentiation of the more mature subpopulations from the thymus. Interestingly, this is in opposite to the LM containing the α2 chain, which are located in the subcapsular region and functionally seems to affect more immature subpopulations.

Gene expression for α3, β3, and γ2 LM chains (which form LM-332) was also detected in the mouse thymus. Nevertheless, at variance with the pattern reported for humans, in the mouse thymus, *in situ* hybridization together with immunohistochemical studies revealed a more restricted distribution of this LM isoform, only in the outer cortex of the thymic lobules (69). The discrepancies concerning LM-332 distribution in the thymus of these species remain to be elucidated. They can be real biological differences, or they can derive from differences in the reagents applied. In any case, using anti-LM-332 blocking antibodies in fetal thymus organ cultures, the authors found an arrest in T-cell development at the DN stage of differentiation, thus unraveling the role of this isoform in thymus physiology (69).

Laminin isoforms containing α4 and α5 chains are the main isoforms found in the basal membranes of the endothelium (70). Accordingly, in the human thymus, α4 LM chain is restricted to the endothelium, as defined by anti-PECAM staining, with the isoforms 411 and 421 present in the blood vessels (29). However, PCR analysis of TEC and TNC bulk extracts in culture suggests the presence of LM containing the α4 chain (57), also indicating that particular culture conditions may modify the expression of LM chains.

Laminin-511 is widely distributed in the body and, in the basement membrane of the blood vessels, LM-511 and LM-521 are normally associated with the isoform 411 (70). In the thymus, the α5 LM chain was observed in the subcapsular epithelium of the thymic lobules and in the thymic blood vessels, together with LM-411. Functionally, *in vitro* experiments showed that the thymocytes binding to LM-511 was mediated by the integrin α6β1, with a preferential adhesion of DN thymocytes to LM-511/LM-521 (29).

If in one hand LM isoforms containing the α2, α4, and α5 chains can be detected in thymic blood vessels, the expression of the isoform LM-332 (containing the α3 and γ2 chains) seems to be a main feature of mTEC (29, 56). It is thus conceivable that distinct LM isoforms play specific roles in the intrathymic T-cell differentiation and migration.

Overall, the RT-PCR analysis of human LM isoform gene expression together with immunohistochemical data on tissue sections and cultured TEC and TNC showed that genes coding for LM components are actively transcribed both *in situ* and in TEC cultures, and actually the corresponding polypeptides are assembled to form the respective isoforms (29, 56, 57). It is important to mention that the studies employing PCR from thymus bulk extracts suggest the presence of LM-211, LM-332, LM-411, and LM-511, which are already described *in situ*, using specific antibodies for the LM chains (see Table 2). Additionally, these works suggest the presence of LM-311 and γ3-containing LM isoforms (29, 57). Yet, these studies are essentially based on the thymus sections from infants, and the knowledge on fetal as well as aging thymus has not been settled yet.

**Table 2:** ***In situ* intrathymic expression of laminin isoforms^a^**.

Laminin isoforms^b^	Subcapsular cortex	Inner cortex	Medulla	Perivascular
LM-211	****+****	****−****	****−****	****+****
LM-221	****+****	****−****	****−****	****+****
LM-332	****+****	****−****	****+****	****−****
LM-411	****−****	****−****	****−****	****+****
LM-421	****−****	****−****	****−****	****+****
LM-511	****+****	****−****	****−****	****+****
LM-521	****+****	****−****	****−****	****+****

*^a^Data compiled from Ref. (29, 31, 55, 56, 68, 69)*.

*^b^We did not find any published report concerning the intrathymic localization of other LM isoforms, despite some LM chains were detected in thymic bulk extracts*.

Among the various integrin-type LM receptors described in the thymus, VLA-6 (α6β1 or CD49f/CD29) is the most studied; being expressed by developing thymocytes, in their various CD4/CD8-defined subsets, as well as thymic microenvironmental cells, particular TEC. Nevertheless other LM receptors, such as α3β1 and α6β4, are also constitutively expressed by developing thymocytes and TEC (30, 57, 63, 71).

The intrathymic distribution of LM isoforms in normal conditions is summarized in Table 2, whereas the expression of some integrin-type LM receptors by thymic cells is seen in Table 3.

**Table 3:** **Expression of integrin-type laminin receptors by developing thymocytes and thymic microenvironmental cells^a^**.

Laminin receptor^b^	CD4/CD8-defined developing thymocytes^c^				Thymic microenvironmental cells		
	DN	DP	CD4 SP	CD8 SP	TEC	TDC	Endo
α1β1	−	−	−	−	+	ND	+
α2β1	−	−	−	−	+	+	+
α2β2	ND	ND	ND	ND	+	ND	ND
α3β1	+	−	+	+	+	ND	+
α6β1	+	+	+	+	+	ND	ND
α6β4	+	−	+	+	+	ND	ND
α7β1	ND	ND	ND	ND	+	ND	ND

*^a^Data compiled from Ref. (63, 71–75)*.

*^b^We did not find any published report concerning the intrathymic expression of the following laminin-binding integrins: α9β1, αvβ3, αvβ5, αvβ8, and αMβ2*.

*^c^DN = CD4^−^CD8^−^; DP = CD4^+^CD8^+^; CD4 SP = CD4^+^CD8^−^; CD8 SP = CD4^−^CD8^+^*.

*TEC, thymic epithelial cell; TDC, thymic dendritic cell; Endo, endothelium; ND, not determined*.

## Role of LMs in Thymocyte Migration

### LM-Mediated Interactions as a Vector Influencing Thymocyte Migration

Migration within a solid tissue requires transient adhesion of migrating cells on the cellular and ECM substrates. Accordingly, adhesion assays, as well as migration assays using *transwell* migration chambers have been extensively used to test whether LMs are involved in thymocyte migration. Moreover, we have used TNC to study migration of developing thymocytes in the context of the tridimensional structure of these lymphoepithelial complexes (76). TNC can be isolated from the thymus of various species and contain one TEC that harbor a variable number of thymocytes, mostly immature DP cells. These complexes can be settled in culture, where they progressively release thymocytes. Additionally, TNC-derived epithelial cells can be let in contact with immature thymocytes and re-form the classical TNC lymphoepithelial structures. Accordingly, evaluation of thymocyte release and reconstitution of TNC complexes may be applied to approach the exit and entrance of thymocytes in the epithelial niche.

We have showed that thymocyte adhesion onto TEC monolayers is partially mediated by LM/VLA-6 interactions, being largely impaired in the presence of anti-CD49f monoclonal antibodies (63). Furthermore, when we treated mouse or human TEC with different stimuli that enhance LM production and LM receptor expression, we found an increase of thymocyte adhesion; an effect that could be prevented by anti-LM or anti-CD49f antibodies (63, 77–79). This interaction is also blocked by monoclonal antibodies specific for integrin α6β1 and its LM ligand LM-211 (57). This is similar to the previous studies performed in the mouse thymus showing that the thymocyte adhesion onto LM-211 could be abrogated by anti-CD49f or anti-CD29 antibodies (recognizing α6 and β1 integrin chains, respectively) (30). LM-111 as well as LM-211 can also modulate thymocyte trafficking in TNC complexes, stimulating both the entrance and exit of thymocytes in cultures of the TNC lymphoepithelial complexes, and such migratory patterns could be blocked with anti-CD49f antibodies recognizing α6 integrin chain (19, 63, 77).

We have proposed a model termed multivectorial thymocyte migration. Accordingly, a vector represents, as in Physics, a force that has magnitude and direction (and can be associated to other vectors) acting upon an object. In this sense, the oriented movement of developing thymocytes – resulting from cell velocity and displacement in a given moment and in a particular thymic zone – might be a resulting vector from individual migrating vectors, which represent the various ligand–receptor interactions that influence thymocyte migration (18, 80, 81). This model can be seen in Figure 1B. Moreover, using *ex vivo* migration assays, we found that a given cell migration-related ligand/receptor pair interaction can modulate another pair (82–84), thus rendering the model still much more complex.

Laminin-mediated interactions can be placed as an individual vector in the multivectorial model (or group of vectors depending on each isoform), inducing thymocyte migration and consequently differentiation, contributing to the final resulting vector, alone or combined with other molecules, as we further discuss below. This is illustrated in Figure 1B.

It is well described that LM (in this case LM-111) induces mouse thymocyte haptotaxis in *ex vivo* assays using freshly isolated thymocytes (18, 82, 83). Also, experiments using mouse LM-111 revealed that LM-mediated interactions are relevant for the entrance of T-cell precursors into the thymus (19, 85), for the migration of developing thymocytes, both in mice and humans (57, 68, 86), as well as in peripheral lymphoid organs (87, 88).

Data from mouse and human models revealed that LM-211 also exerts a haptotactic effect upon normal thymocyte migration, as revealed by *ex vivo* transmigration assays, as well as by *in vitro* thymocyte entrance and exit of TNC lymphoepithelial complexes. Again, this interaction is also blocked by monoclonal antibodies specific for integrin α6β1 and its LM ligand LM-211 (57). This is similar to the previous studies performed in the mouse thymus showing that the thymocyte adhesion onto LM-211 could be abrogated by anti-CD49f or anti-CD29 antibodies (30). Additionally, in the human model, it was showed that thymocyte adhesion can be impaired by anti-α3 integrin chain antibodies (57), strongly indicating that LM-211 can modulate thymus physiology by using at least two distinct LM-binding integrins.

**Figure 2:**
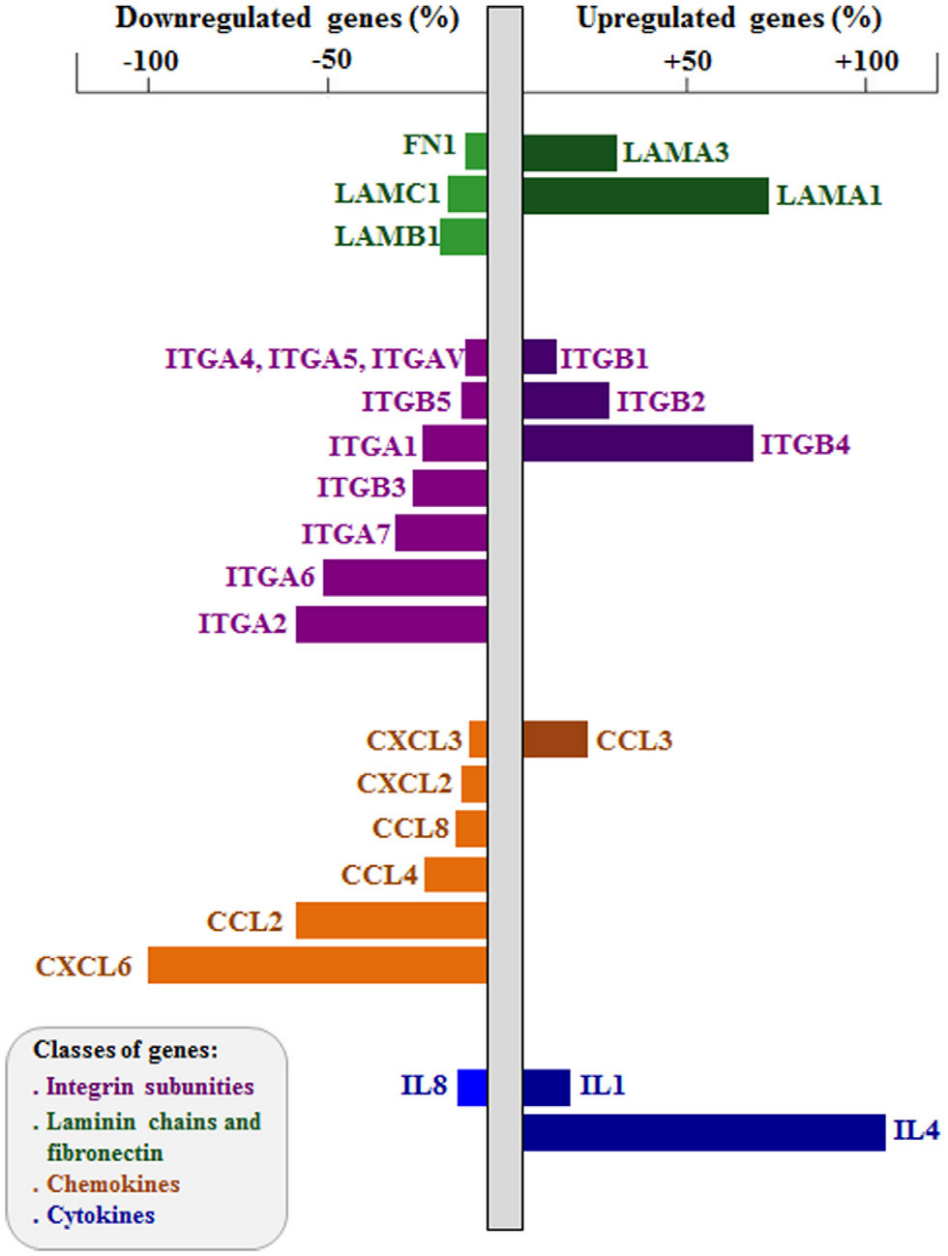
**Effects of integrin α6 knockdown on cell adhesion and migration-related genes of human thymic epithelium**. The figure shows extracellular matrix and chemokine ligand genes up- and downregulated after integrin α6 (ITGA6) knockdown by small interference RNA (siRNA) strategy. Gene expression was defined using PCR arrays, comparing ITGA6 siRNA-treated thymic epithelial cells (*n* = 5) with control siRNA cells (*n* = 5). Bar colors indicate different group of genes (LM chains and FN in *green*, integrin sub-unities in *purple*, chemokines in *orange* and cytokines in *blue*). Right bars are upregulated genes and left bars are downregulated genes, both showed in percentage. Two-tailed unpaired Student’s *t*-test was applied, and the differences showed were all significant. Data derived from Ref. (72).

More recently, we applied the RNA interference (RNAi) approach to study the role of integrin-type ECM receptors upon the thymic epithelium and the resulting interactions with developing thymocytes (72, 89). Modulation in both gene and protein expressions affecting LM-mediated interactions has been reported influencing thymocytes. For example, RNAi-induced knockdown of the ITGA6 gene in cultured human TEC induced a significant decrease in CD49f membrane expression, with consequent decrease in the adhesion of these cells to LM and to cultured TEC. Moreover, we found up- and downregulation of a large number of cell migration-related genes, including those encoding LM chains, integrin sub-unities, as well as chemokine and cytokines; some of them are illustrated in Figure 2 (72). In this case, genetic modulation provided important information on the regulatory relation between the α6 integrins and thymic epithelium adhesion and migration genes. These data clearly show that downregulation of ITGA6 gene in the human thymic epithelium triggers a complex cascade of effects upon the expression levels of several other cell migration-related genes, including ECM and chemokine ligands and receptors. Taken together, these data unravel the concept that the expression of genes involved in controlling of thymocyte migration by the thymic microenvironment should be regarded as complex networks, so that a defect in the expression of one single gene may reflect in an amplified cascade with functional consequences for TEC adhesion onto the natural ligand and potential consequences upon the normal patterns of TEC/thymocyte interactions.

### Other Cell Migration-Related Vectors Act Synergistically with LM on Thymocyte Migration

**Figure 3:**
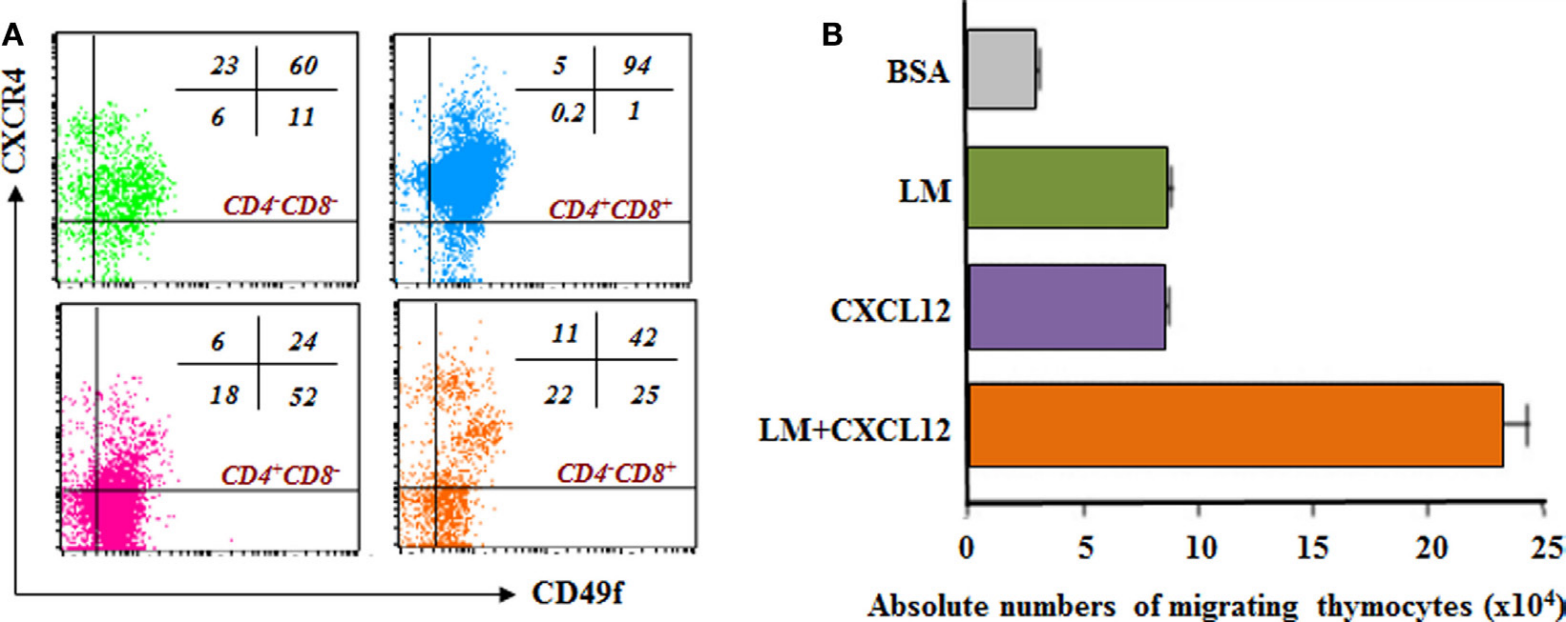
**Laminin receptor expression and laminin-driven migration of mouse thymocytes: relationship with CXCL12/CXCR4 interactions**. **(A)** depicts a dot plot derived from four-color cytofluorometric immunostaining of mouse thymocytes. The panel shows the simultaneous membrane expression of CD49f (α6 integrin chain) and CXCR4, the CXCL12 chemokine receptor, in the four CD4/CD8-defined thymocyte subsets. In this panel, it is clear that in all immature and thymocyte thymocyte subpopulations, there are cells expressing both receptors. The numbers seen in each dot blot represent the percentages of cells expressing either CD49f and/or CXCR4 in each CD4/CD8-defined thymocyte subset. **(B)** reveals that thymocytes migrate toward CXCL12 and LM-111, as compared to bovine serum albumin (BSA), used as negative control. Interestingly, when migratory stimuli were applied simultaneously, the resulting migration was higher than the sum of each stimulus used alone, strongly indicating synergy between LM and CXCL12 to stimulate cell migration. Data derived from Ref. (19).

One new interesting topic of study approaches the possibility that other factors could act synergistically with LM and significantly influence thymocyte migration. In this context, LM-111 can enhance the chemotactic activity of chemokines when both molecules are placed in combination in transmigration chambers. The CXCL12 chemokine *per se* also induces thymocyte migration, but when combined with LM a synergic migratory response can be observed in different thymocyte subpopulations, mainly the immature DP cells Figures 3A,B (90).  Interestingly, a synergic effect was also observed for LM combined with other chemokines as CCL22 and CCL19, but in this case the more responsive subpopulations were mature CD4 and CD8 SP thymocytes.

These observations suggest that LM can enhance responsiveness to chemokines but depending on the combination of molecules, the response is more evident in specific thymocyte subsets. Nevertheless, these effects were measured using LM-111, and so far we do not know whether other LM isoforms act similarly. Nevertheless, these data point to a possible bidirectional influence between a given migration vector and LM-mediated interaction upon thymocyte migration. Yet, further studies are warranted to mechanistically approach such a hypothesis.

### Hormonal Control of LM-Mediated Thymocyte Migration

Laminin and VLA-6 expression in the thymus can be upregulated by hormones such as the growth hormone (GH) (88). We initially showed that GH treatment of human TEC enhances LM production, the expression of LM, with consequent increased adhesion of thymocytes to treated TEC, and also the release of thymocytes from TNC; effects that could be abrogated by anti-LM and anti-LM receptor antibodies (79). *In vivo* studies showed that thymocytes from GH-transgenic mice, or from control mouse thymus injected with GH, migrate more through LM, but not through fibronectin (FN), when comparing to controls (82, 91). The combination of LM with CXCL12 synergized migratory responses, and this phenomenon could be observed in all thymocyte subpopulations. Interestingly enough, treatment with pertussis toxin (which catalyzes Gαi ADP-rybosilation, therefore resulting in signaling inhibition of G protein-coupled receptors, including chemokine receptors) prior to *ex vivo* migration assays blocked the migration of both control and GH-transgenic mouse thymocytes but did not change the profiles of LM-triggered thymocyte migration. However, when thymocytes from GH-transgenic mice were preincubated with anti-VLA-6-blocking mAb (that recognizes the CD49f chain) and then subjected to CXCL12 stimulation, a decrease in the numbers of migrating cells is observed (82). These results reinforce the idea of a crosstalk between integrins and chemokine receptors and the fact that the relative influence of VLA-6 on the CXCR4 signaling pathway seems to be more relevant than the reverse situation.

Triiodothyronine (T3), a thyroid hormone critical for the regulation of body metabolism, growth, and development, also exerts several effects on thymus physiology. Mice treated with T3 exhibited an increase in thymus weight and cellularity and an enhancement in the intrathymic expression of ECM and ECM receptors on thymocytes, including LM and VLA-6, respectively (92–94). Consequently, after short- and long-term T3 treatments, migration of thymocytes through LM is increased in *ex vivo* assays, proportionally to the treatment. Moreover, T3 stimulates thymocyte egress to peripheral lymphoid organs. Recent thymic emigrants (RTE) were evaluated by flow cytometry in the spleen, subcutaneous, and mesenteric lymph nodes 16 h after intrathymic FITC injection, and it was observed that T3 treatment changed the distribution of RTE among lymphoid organs, directing cells to lymph nodes instead of the spleen (95). These findings indicate that T3, by enhancing migratory capacity of thymocytes in response to LM, can redirect lymphocyte homing in the periphery.

Glucocorticoid (GC) hormones also regulate intrathymic LM-mediated interactions. Mice injected with GCs exhibit a rapid thymocyte depletion, which is paralleled by an enhancement of LM deposition within both cortical and medullary regions of the thymic lobules. Such an effect is not only due to the atrophy of the organ, since treating cultured TEC with dexamethasone also enhances LM production (52). Additionally, developing thymocytes from GC-treated mice exhibit a higher membrane expression of the CD49f chain of LM receptor (63). Moreover, GC treatment on cultured TEC enhances LM production, increases thymocyte adhesion, as well as the release of thymocytes from TNC complexes; effects that can be reversed by anti-CD49f antibody treatment (63). Overall, these data showed that GC is able to modulate cell migration-related LM-mediated interactions in the thymus. Nevertheless, it is to be determined if GC *per se* can directly induce thymocyte migration or act via the effect on TEC.

## Other Molecules Controlling LM-Mediated Migration

The expression of some molecules initially described in the nervous system, such as neuropilins (NRPs) and ephrins, can also be observed in the thymus. Interestingly, these molecules were also shown to play a role in thymocyte migration and in modulating LM-induced haptotactic responses.

Neuropilins are transmembrane glycoprotein receptors for secreted class 3 semaphorins (Semas) and some vascular endothelial growth factor isoforms (96). Several NRP/Sema receptor/ligand pairs are expressed in the mouse and human thymuses, in both lymphoid and non-lymphoid compartments (97). The expression patterns of NRPs and Semas, such as NRP1/Sema3A, suggests their involvement in numerous cell processes in the thymus, including thymocyte adhesion and migration. Sema3A treatment, for example, decreases the adhesion of human NRP1^+^ thymocytes on TEC monolayers (83). Moreover, Sema3A exerts a dose-dependent chemorepulsive effect on human thymocytes, and it inhibits LM-induced thymocyte migration (83). Yet, this inhibitory effect is not restricted to LM, since Sema3A treatment also abrogates FN or CXCL12-driven migration of all CD4/CD8-defined thymocyte subsets (95).

Ephrins are also expressed in numerous cell types, including TEC and thymocytes, and bind the Eph receptor family, which corresponds to the largest family of tyrosine-kinase receptors in animal cells (98). In the thymus, it has been demonstrated that Eph/Ephrins signaling is relevant for cell migration, including the entry of bone marrow progenitor cells, migration of thymocytes within the organ and thymocyte export, triggered by chemokines and/or ECM proteins, including LM-111. Eph stimulation by ephrin B1 inhibits LM-111- and FN-driven migration responses as well as CXCL12-, CCL21-, and CCL25-induced chemotaxis of both bone marrow progenitors and thymocytes from normal mice (81, 85). The fact that LM-111 is involved in the entrance of T-cell precursors into the developing thymus is in keeping with previous data showing that anti-CD49f antibodies largely reduced the number of thymocytes in a mouse model of thymus colonization by T-cell precursors (99). Yet, we do not know whether other LM isoforms are biologically active in promoting these effects.

Noteworthy, the prion cellular protein (PrP^C^) can also be found in the nervous and immune systems and can bind LM through its γ1 chain (100–103). In the thymus, PrP^C^ is physiologically expressed by mouse thymocytes and TEC. Although PrP^C^-deficient mice do not exhibit major changes in thymus physiology, PrP^C^ overexpression has a major influence in thymocyte development, since PrP^C^ transgenic mice show a severe thymic hypoplasia with thymocyte differentiation being largely blocked at immature stages (104). Moreover, the remaining immature thymocytes present impaired LM-driven migration, and this is paralleled by the fact that the number of CD4^+^ and CD8^+^ T cells in peripheral lymphoid organs is largely diminished (105). All together, these findings indicate that overexpression of PrP^C^ generates a multifaceted disturbance in T-cell development, and change, at least via LM-driven interactions, the general migration pattern of developing thymocytes, including their export from the organ.

Despite not being shown to directly interact with LM in the thymus, other ECM associated molecules that mediate thymocyte interactions via integrin-type LM receptors have been reported. Recently, the LM-related molecule named netrin-1, also initially described in the nervous system, was found to be expressed in thymic stromal cells and also in anti-CD3- or IL-7-stimulated thymocytes (106). Additionally, *in vitro* assays demonstrated that thymocytes strongly adhere to netrin-coated surfaces, in a manner partially dependent on α6β4 integrin, and netrin was shown to potentiate CXCL12-driven chemotaxis of thymocytes (106).

Another matricellular protein called Cyr61/Ccn1, which binds α6β1 integrin (107), is produced by thymic microenvironmental cells (108), with TEC as the main source, and was shown to enhance both TEC proliferation and TEC–thymocyte interactions (109).

### Expression of LM and Its Possible Role on Thymocyte Migration in Disease

#### Infectious Diseases

In some pathological conditions, the expression/deposition of LM and LM receptors in the thymus can be modulated. These alterations can, in turn, alter thymocyte migration. This is the case of the *Trypanosoma cruzi* infection, the experimental model of Chagas disease. Thymuses from *T. cruzi* acutely infected mice present a severe atrophy, which is characterized by death of DP thymocytes by a caspase-dependent apoptosis mechanism (110–113). In parallel, we described a premature exit of immature DN and DP cells (which may have not been subjected to intrathymic selection processes) to peripheral lymphoid organs, and that persists in the chronic phase of Chagas disease, both in mice and humans (111, 114, 115). In keeping with these data is the fact that the release of immature thymocytes from TNC is enhanced after *in vivo* or *in vitro T. cruzi* infection (116). Considering that immature DP T cells abnormally released from the thymus have an activated phenotype (114), it is possible that thymocyte migration disturbances could be partially responsible for the tissue lesions observed in the pathophysiology of the disease. Interestingly, an enhanced deposition of LM is observed in the thymus of infected mice, as well as an increased expression of VLA-6 on both immature and mature thymocytes and of LM production by TEC (111, 117). Nevertheless, whether LM-mediated interactions play a role in the export of immature thymocytes is an issue to be determined. In any case, it does not seem to be dependent on the rise of circulating GC hormones seen in the acute infection (115, 118, 119), since *in vivo* GC treatment in normal mice does not induce export of these immature thymocytes (111).

The thymus is also a target organ in *Plasmodium berguei* infection, with induction of organ atrophy, apoptosis-induced death of DP thymocytes, enhanced chemokine (CXCL12) and ECM contents (LM and FN), and modulation of ECM and chemokine receptor expression on thymocytes (120). Thymocytes from infected animals express lower amounts of VLA-6, mainly the immature DP ones. Conversely, the expression of chemokine receptors is augmented when comparing to non-infected animals. In this case, DP cells, which express lower amounts of VLA-6, migrate less through LM, while mature CD4 and CD8 SP cells migrate more. When combining LM with chemokines, it was observed that LM enhanced CXCL12 and CCL25-induced migration of mature thymocytes from infected mice, suggesting that alterations in LM and chemokine expression, as well as their respective receptors, can modulate thymic function and export of mature lymphocytes during *P. berguei* infection.

Although the results are clear, it is important to have in mind that these functional studies have been conducted using mouse LM-111, and we do not know whether similar migratory responses occur toward other LM isoforms, an issue that is worthy to be investigated.

In a second vein, it should be pointed out that the thymus is a target organ in several viral, bacterial, and parasitic diseases [reviewed by Nunes-Alves et al. (121) and Savino (122)], and studies should be performed in various human and animal models of disease in order to define if the alterations described above are specific to given infectious agents, or rather universal.

#### Type 1 Diabetes

The non-obese diabetic (NOD) mouse spontaneously develops type 1 autoimmune diabetes, which is mediated by T-cell-dependent destruction of pancreatic insulin-producing beta-cells (123). Studies in prediabetic animals revealed that the thymus of NOD mice present several alterations, including a higher LM deposition and the formation of giant PVS filled with LM (intermingled with other basement membrane proteins, such as type IV collagen and FN) and mature thymocytes that accumulate within the organ (18, 124–126).

Unfortunately, these immunolocalization studies have been conducted using anti-LM polyclonal reagents, and it remains to be determined if the intra-PVS LM network is formed by a particular LM isoform.

Thymocytes from NOD mice present lower levels of membrane VLA-5 (a FN receptor), but a higher VLA-6 expression when comparing with C57BL/6 mice (18, 126). This higher expression was accompanied by enhanced LM-driven migration and LM-driven upregulation of CXCL12-induced migration of thymocytes. Interestingly, an opposite effect was observed with FN. The defect in VLA-5 expression was accompanied by a diminished CXCL12-induced migration through FN-coated membranes (18). As thymocytes accumulate in thymic PVS, it is conceivable that, although thymocytes interacting with LM are able to migrate toward chemokines, a preponderant role is played by the aberrant VLA-5 expression and the consequent decreased FN-driven migration capacity of NOD thymocytes. All together, these findings indicate that, in the NOD mouse thymus, different individual vectors, such as those mediated by FN and LM interactions, regulate positively or negatively the resulting migratory response.

Another relevant point to have in mind is that, as mentioned before, these studies have been performed in prediabetic NOD mice, and it has not been defined if the same changes remain after overt diabetes has been settled in these animals.

In this respect, we have recently evaluated normal mice turned diabetic after a single injection of alloxan, a drug known to destroy insulin-producing cells in the pancreas. The distribution and density of LM in thymuses from diabetic animal group (3 days following alloxan injection) was increased in both cortical and medullary regions of the thymic lobules, as compared to controls. By contrast, there was a decrease in VLA-6 expression in developing thymocytes from diabetic mice, although the migratory response of these thymocytes to LM was not altered, in the presence or absence of CXCL12 (127).

Overall, the data summarized above indicate that LM receptor expression as well as migratory responsiveness to LM may change before versus after overt diabetes. Nevertheless, this hypothesis should be taken with precaution since the experimental animals used were different.

Lastly, whether or not thymocytes from NOD or alloxan-treated animals respond similarly to LM isoforms other than LM-111 has not been investigated so far, and is obviously a missing point that deserves to be investigated.

## Concluding Remarks

Cumulative data summarized herein clearly show that LMs and their integrin-type receptors are constitutively expressed in the thymus, and that LM-mediated interactions are comprised within the complex ligand/receptor molecular network that ultimately guide thymocytes in a fine-tuned oriented movement of developing thymocytes throughout the thymic lobules. If we use the multivectorial concept of intrathymic T-cell migration, it is clear that the LM-mediated interactions can be considered one vector (or group of vectors). Furthermore, this vector can be controlled by other vectors (such as those represented by semaphorin/NRP and Eph/Ephrin interactions) and can also influence other vectors, as that mediated by CXCL12/CXCR4, and possibly many others.

It should be noted however that several issues should be elucidated. For instance, if there is a natural mutant *dy/dy* mouse, where thymocyte development is severely affected, studies on the thymus of genetically engineered animals, already produced by either conventional or conditional knockout strategies for other LM or LM receptor chains, have not been conducted so far. Such studies certainly represent an important open avenue for future research. Similarly, although we have silenced the ITGA6 gene in human-cultured TEC by specific small interference RNA and did find modulation of a large number of cell migration-related genes and a downregulatory effect upon TEC adhesion (72), this strategy has not been used *in vivo*, after direct injection within the thymus.

Another aspect deserving to be experimentally approached is whether or not there is functional redundancy of distinct LM isoforms in terms of the effects upon thymocyte migration.

Developing these issues will provide important clues for better define the precise role of LM-mediated interactions upon intrathymic T-cell migration and development in health and disease.

## Conflict of Interest Statement

The authors declare that the research was conducted in the absence of any commercial or financial relationships that could be construed as a potential conflict of interest.
